# Reply to Improda, N.; Ballarin, G. Body Composition in Paediatric GLP-1 Receptor Agonist Therapy. Comment on “Zaitoon et al. Beyond Weight Loss: Optimizing GLP-1 Receptor Agonist Use in Children. *Children* 2025, *12*, 1427”

**DOI:** 10.3390/children13050608

**Published:** 2026-04-28

**Authors:** Hussein Zaitoon, Aimee D. Wauters, Luisa M. Rodriguez, Jane L. Lynch

**Affiliations:** Division of Pediatric Endocrinology and Diabetes, University of Texas Health Science Center, San Antonio, TX 78229, USA; zaitoon@uthscsa.edu (H.Z.);

Dear Editor,

We thank the authors for their insightful commentary on our paper [1,2] and agree that the physiological context of growth and puberty limits direct extrapolation of adult body composition data to pediatric populations. We also acknowledge the findings cited from Apperley et al., demonstrating reduced fat mass with a numerical increase in fat-free mass in adolescents treated with liraglutide [3]. However, it is important to note that the magnitude of weight loss in this study was modest and the duration was short. In this context, the absence of measurable changes in lean mass is not unexpected.

While emerging clinical experience suggests that greater weight loss may occur with newer GLP-1RA therapies, particularly with agents such as semaglutide, systematic pediatric data characterizing these effects remain limited. Based on adult data, greater total weight reduction is consistently associated with a higher proportion of lean mass loss (approximately 20–40%) [4,5]. However, these findings should be interpreted with caution when considering pediatric populations. Therefore, while early pediatric data are reassuring, the potential impact on lean mass and muscle mass may become more clinically relevant as the magnitude and duration of weight loss increase.

For this reason, our inclusion of adult body composition data was intended to highlight a broader and potentially underrecognized concern: that significant weight loss, particularly when rapid or sustained, may be associated with reductions in lean mass and could have implications for overall musculoskeletal health. At present, this concern remains biologically plausible and clinically relevant, but not definitively established in pediatric populations. Given the limited pediatric-specific evidence, we believe this issue warrants careful consideration rather than reassurance and is best approached as a precautionary concern.

We agree with the authors that methodological challenges and knowledge gaps remain substantial. Current pediatric studies largely focus on anthropometric outcomes, with limited evaluation of body composition, including differentiation between lean mass, skeletal muscle mass, and functional musculoskeletal outcomes [6,7,8]. As GLP-1RA use expands in children and adolescents, prospective pediatric studies incorporating validated body composition and functional assessments will be essential to better define these potential effects.

We appreciate the authors’ contribution to this important discussion and share their view that further research is urgently needed to guide safe and effective use of GLP-1RA therapy in pediatric populations.
